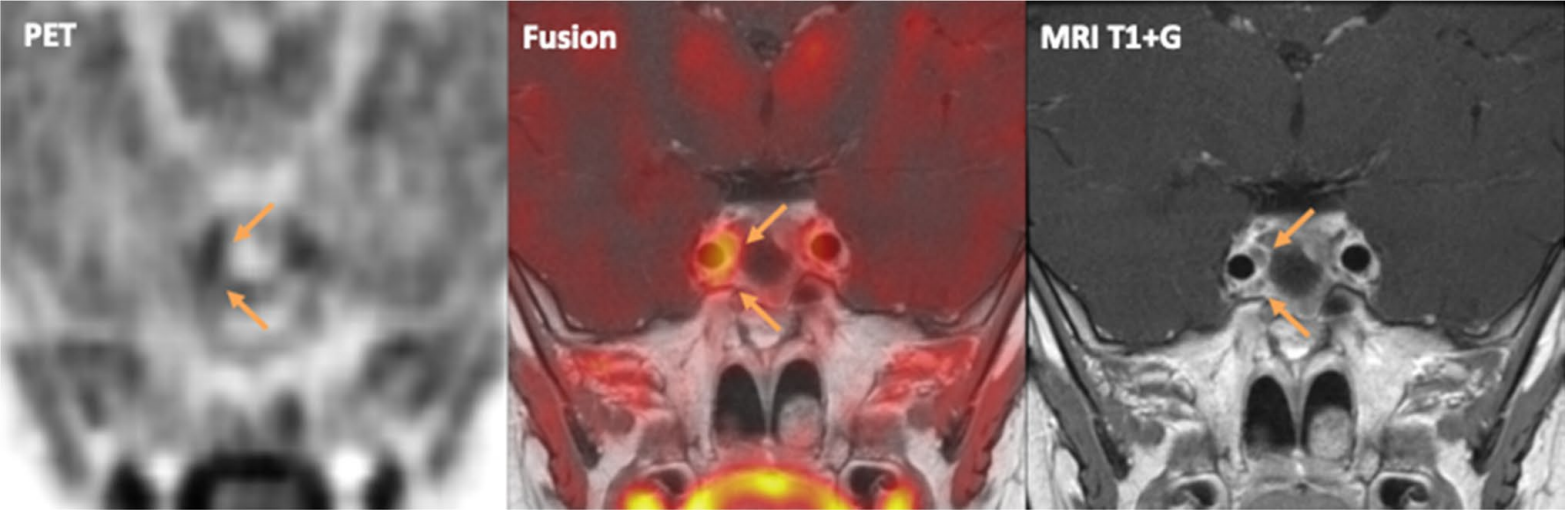# Positive [^18^F]fluoroethyltyrosine PET/MRI in suspected recurrence of growth hormone–producing pituitary adenoma in a paediatric patient

**DOI:** 10.1007/s00259-021-05458-1

**Published:** 2021-07-05

**Authors:** Sophie E. M. Veldhuijzen van Zanten, Sebastian J. C. M. M. Neggers, Roelf Valkema, Frederik A. Verburg

**Affiliations:** 1 Department of Radiology and Nuclear Medicine, Erasmus MC, Dr. Molewaterplein 40, 3015 GD Rotterdam, The Netherlands; 2 Department of Medicine, Section Endocrinology, Rotterdam, the Netherlands

A 6-year-old boy was referred because of a suspected recurrence of a growth hormone (GH)–producing pituitary macro-adenoma, which initially expanded from the sella turcica to the suprasellar and right parasellar region. Over the course of 2.5 years, the patient had undergone three transsphenoidal resections after which biochemical values normalized. At time of referral, the patient again experienced symptoms of localized headache and increasing GH and IGF-1 serum levels. Repeated MRIs were obtained under a dedicated pituitary imaging protocol, but paediatric neuroradiologists were unable to conclusively differentiate between recurring adenoma and post-operative granulation tissue.

As one report of the incidental finding of a macro-adenoma on 18F-fluoroethyl-l-tyrosine ([^18^F]FET) PET has been described [1], we decided to perform a [^18^F]FET PET/MRI in our patient, in an attempt to confirm the suspected recurrence. [^18^F]FET is a non-natural amino acid transported by the amino acid transport system L (LAT), of which particularly the LAT1 isoform is expressed in a variety of tumour cells. A recent study in rat showed higher LAT1 gene levels in GH-producing pituitary tumour cells compared to normal pituitary tissue [2].

In our case, PET/MR-images revealed increased [^18^F]FET uptake caudomedially of the right internal carotid artery (Fig. 1A/B). The uptake correlated with two nodular lesions of approximately 3 and 5 mm diameter (Fig. 1C), which were indeed progressive in size over time. This confirmed recurrent adenoma foci, and establishes the usability of [^18^F]FET PET/MRI for imaging of pituitary adenoma. Our patient was referred for radiotherapy, since a fourth resection was not considered possible.